# Genome-wide identification, evolution and expression analysis of bone morphogenetic protein (BMP) gene family in chinese soft-shell turtle (*Pelodiscus sinensis*)

**DOI:** 10.3389/fgene.2023.1109478

**Published:** 2023-02-01

**Authors:** Luo Lei, Junxian Zhu, Chen Chen, Yongchang Wang, Congcong Wu, Ming Qi, Yakun Wang, Xiaoli Liu, Xiaoyou Hong, Lingyun Yu, Haigang Chen, Chengqing Wei, Yihui Liu, Wei Li, Xinping Zhu

**Affiliations:** ^1^ Key Laboratory of Tropical and Subtropical Fishery Resources Application and Cultivation, Ministry of Agriculture and Rural Affairs, Pearl River Fisheries Research Institute, Chinese Academy of Fishery Sciences, Guangzhou, Guangdong, China; ^2^ Wuxi Fisheries College, Nanjing Agricultural University, Wuxi, Jiangsu, China; ^3^ College of Fisheries and Life Science, Shanghai Ocean University, Shanghai, China; ^4^ Zhejiang Fisheries Technical Extension Center, Hangzhou, China

**Keywords:** Pelodiscus sinensis, bone morphogenetic protein, gene family, genome-wide, transcriptome, sex differentiation

## Abstract

**Introduction:** Bone morphogenetic proteins (BMPs) play a crucial role in bone formation and differentiation. Recent RNA-Seq results suggest that BMPs may be involved in the sex differentiation of *P. sinensis*, yet more relevant studies about BMPs in *P. sinensis* are lacking.

**Methods:** Herein, we identified BMP gene family members, analyzed the phylogeny, collinear relationship, scaffold localization, gene structures, protein structures, transcription factors and dimorphic expression by using bioinformatic methods based on genomic and transcriptomic data of *P*. *sinensis*. Meanwhile, qRT-PCR was used to verify the RNA-Seq results and initially explore the function of the BMPs in the sex differentiation of *P. sinensis*.

**Results:** A total of 11 BMP genes were identified, 10 of which were localized to their respective genomic scaffolds. Phylogenetic analysis revealed that BMP genes were divided into eight subfamilies and shared similar motifs (“WII”, “FPL”, “TNHA”, “CCVP”, and “CGC”) and domain (TGF-*β* superfamily). The results of the sexually dimorphic expression profile and qRT-PCR showed that *Bmp2*, *Bmp3, Bmp15l, Bmp5, Bmp6 and Bmp8a* were significantly upregulated in ovaries, while *Bmp2lb*, *Bmp7*, *Bmp2bl* and *Bmp10* were remarkable upregulated in testes, suggesting that these genes may play a role in sex differentiation of *P*. *sinensis*.

**Discussion:** Collectively, our comprehensive results enrich the basic date for studying the evolution and functions of BMP genes in *P*. *sinensis*.

## 1 Introduction

The transforming growth factor-beta (TGF-*β*) superfamily is a large group of extracellular growth factors controlling many aspects of development (Chang, Brown et al., 2002). Researches have shown that a total of 33 members of the TGF-*β* superfamily are identified in Humans, including 10 members of bone morphogenetic proteins (BMPs), 10 members of growth differentiation factors (GDFs), and 13 additional members (Morikawa et al., 2016). As a dominant subfamily of the TGF-β superfamily, the activity of BMPs was initially investigated in the 1960s (Urist, 1965). BMPs play a major role in osteoblast differentiation and bone formation (Lademann et al., 2020; Yang et al., 2021), and are associated with cancer progression, activating the proliferation and increasing the invasiveness of cancer cells (Ehata and Miyazono, 2022). Moreover, BMPs modulate vascular calcification by regulating the phenotypic plasticity of multipotent progenitor lineages (Yang et al., 2021). Simultaneously, BMPs are also involved in inflammation, glucose homeostasis, and energy metabolism (Grgurevic et al., 2016).

Recently, studies have shown that BMP family members have a role in sex differentiation. *Bmp2* and retinoic acid (RA) act collectively to control female germ cell fate in mice (Miyauchi et al., 2017). *Bmp2*, in cooperation with Estradiol-17β, promotes the transformation of somatic cells into primordial granulosa cells and the formation of primordial follicle in mouse ovaries (Chakraborty et al., 2022). In *Gallus gallus domestics*, *Bmp7* exhibits female-biased expression during sex differentiation and was reduced upon aromatase inhibitor-induced female-to-male reversal (Hoshino et al., 2005). Knocking out *Bmp15* in female zebrafish leads to normal development, but these animals’ sexual revert to fertile males during the juvenile period (Dranow et al., 2016). In *Paralichthys olivaceus*, overexpression of *Gdf9* individually or co-expression with *Bmp15* lead to upregulation of most steroid genes, whereas overexpression of single *Bmp15* decreases the expression of steroid genes (Yu et al., 2020). Meanwhile, BMP signaling mediates the differentiation of primordial germ cells of *Gryllus bimaculatus* (Donoughe et al., 2014). Significantly, *Amh*, *Gsdf*, and *Gdf9* share the same domain with BMPs, belonging to the TGF-*β* superfamily (Yu et al., 2020; Pan et al., 2021). In *Epinephelus coioides*, overexpressing *Amh* could lead to a decrease in female-associated genes and estradiol levels and an increase in male-associated genes and testosterone levels, thereby causing female-to-male reversal *in vivo* (Han et al., 2018). Also, *Gsdf* and *Gdf9* are key members of vertebrates’ sex differentiation pathways (Nagahama et al., 2021). Taken together, BMPs are connected to the process of sex differentiation and seem to be conserved in multiple species.

Likewise, sex differentiation in the Chinese soft-shell turtle (*Pelodiscus sinensis*) is the consequence of multi-gene regulation. *P*. *sinensis* is an economically vital aquatic animal, with an annual production of about 330,000 tons (Yearbook, 2019). The growth pattern of *P*. *sinensis* presents sexual dimorphism, with males growing 1.5 times faster than females (Zhou and Zhu, 2011). Consequently, it is necessary to explore the master genes that regulate sex differentiation in *P*. *sinensis*, thereby producing all-male populations. *Dmrt1* is the first major gene identified as involved in male sex differentiation in *P*. *sinensis*, and knockdown of *Dmrt1* causes male-to-female reversal (Sun et al., 2017). Loss of function of *Amh* resulted in complete sex reversal of ZZ embryos, downregulation of *Cyp19a1*, and upregulation of *Sox9* (Zhou et al., 2019). *Rspo1* is essential for female sex differentiation in *P*. *sinensis*, and knocking down *Rspo1* led to a partial female-to-male reversal (Zhang et al., 2021). Particularly, a novel study indicates that *Bmp2* was significantly more expressed in the gonads of female *P. sinensis* than in males by RNA-Seq, suggesting that *Bmp2* may be a potential gene for sex differentiation in *P. sinensis* (Zhu et al., 2022). Nevertheless, further information about BMP family genes in *P. sinensis* remains necessary.

In the present study, we performed genome-wide identification of the members of BMP gene family in *P. sinensis* using bioinformatics methods and analyzed the phylogenetic relationship, collinear relationship, scaffold locations, gene structures, protein structures, transcription factors, and gonad expression profiles of BMP genes. Moreover, qRT-PCR was used to examine the relative expression of BMP gene family members in male and female gonads at different developmental stages of *P*. *sinensis*. This study enriches our knowledge of BMP genes and contributes to further studies on the regulation mechanisms of sex differentiation in *P*. *sinensis*.

## 2 Materials and methods

### 2.1 Genome-wide identification of BMP genes

All genome sequences, protein sequences, and annotation files of *P. sinensis* (https://www.ncbi.nlm.nih.gov/genome/?term=Pelodiscus+sinensis) and *Homo sapiens* (https://www.ncbi.nlm.nih.gov/genome/?term=human) were obtained from the National Center for Biotechnology Information (NCBI) database. The Hidden Markov Model file of BMP genes (PF00019) was downloaded from the Pfam protein family database (http://pfam.xfam.org/family/PF00019) and was used to screen the members of BMP gene family in *P. sinensis* and human genome with HMMER 3.0 software (Finn et al., 2011). Subsequently, the candidate sequences were further analyzed using the NCBI Conserved Domain tool (Marchler-Bauer et al., 2017) and SMART database (Letunic et al., 2021) to proofread the process of the gene family member screening and remove redundant sequences, and the confirmed members of the BMP gene family contained the complete TGF-*β* superfamily domain.

### 2.2 Phylogenetic analysis and collinear analysis of BMP gene family

Amino acid sequences of typical vertebrates were obtained from the NCBI database (http://www.ncbi.nlm.nih.gov), including *Homo sapiens*, *Mus musculus*, *Gallus gallus*, Anas platyrhynchos, *Danio rerio*, *Oryzias latipes*, *Xenopus tropicalis*, *Bufo gargarizans*, *Terrapene carolina triunguis*, *Chelydra serpentina*, *Dermochelys coriacea*, *Mauremys reevesii*, *Caretta caretta*, *Chelonia mydas*, *Mauremys mutica*, and *Chrysemys picta bellii* (All protein accession numbers and sequences used for the analysis were shown in Annex 1). Multiple sequence alignment of full-length proteins among species was assessed using MUSCLE (Edgar, 2004), and an unrooted phylogenetic tree was constructed using the Maximum Likelihood method with a Jones-Taylor-Thornton (JTT) + Gamma Distributed (G) model and 1,000 bootstrap replications using MEGA 7 software (Kumar et al., 2016). The phylogenetic tree was visually improved using the online website ChiPlot (https://www.chiplot.online/). Collinear analysis was performed among human, chicken, *P. sinensis*, and zebrafish using NCBI genome browser (https://www.ncbi.nlm.nih.gov) and Ensembl (https://useast.ensembl.org/index.html).

### 2.3 Analysis of scaffold location, gene structure, conserved motif, and domain

First, BMP genes were mapped to the scaffold locations, and a gene density file of each scaffold was generated according to the genome annotation files. TBtools as a visualization tool to display BMP genes structures (Chen et al., 2020). The protein sequences of BMP genes were submitted to MEME Suite database for predicting conserved motifs, and the motif numbers were searched until exceeding the default thresholds (Bailey et al., 2015). Meanwhile, the NCBI Conserved Domain tool was used to screen the conserved domains of BMP genes with the default parameters (Marchler-Bauer et al., 2017). The visualization of the conserved motifs and domains of BMP genes were applied by TBtools software.

### 2.4 Multiple sequence alignment and prediction of protein structure

The amino acid sequences of conserved domains, from the TGF-*β* superfamily, of BMP genes were obtained by SAMRT (Letunic et al., 2021), and BioEdit software was utilized for multiple sequence alignment. Meanwhile, WebLogo (Crooks et al., 2004) was used to show the characteristics of amino acid sequences. The prediction of protein secondary and tertiary structures were performed using SOPMA (Geourjon and Deléage, 1995) and SWISS MODLE (Waterhouse et al., 2018), respectively.

### 2.5 Analysis of transcription factors

TBtools software was applied to extract a 2-kilobase (kb) promoter region upstream of the transcription start site of the BMP genes. Transcription factors were predicted by JASPAR (Castro-Mondragon et al., 2022). In short, all promoter sequences were used to search BMP genes sites with all available matrices in the taxon of vertebrates *via* the Scan tool and a relative score cutoff of 0.8 was used to identify putative sites. TBtools was used to map the transcription factors to the 2-kb promoter region of BMP genes.

### 2.6 Analysis of sexually dimorphic expression profiles of BMP genes based on transcriptomic data

The transcriptomic data of the gonads in *P. sinensis* was downloaded from NCBI database of Zhu et al. (2022); SRA accession: PRJNA838782). Trimmomatic (Bolger et al., 2014) was used to remove low-quality reads and adapters, and Hisat2 v2.1.0 (Kim et al., 2015) was used to for mapping to the reference genome of *P. sinensi*s. The expression levels of BMP genes were calculated and normalized with the fragments per kilobase million (FPKM) values, using StringTie (v2.2.0) with default parameters (Pertea et al., 2016). Then, the differentially expressed levels of mRNAs were evaluated using edgeR (Robinson, McCarthy, and Smyth 2010)**.** And the screening criteria were a fold change ≥2 and *p <*0.05. Finally, a heatmap was generated using TBtools.

### 2.7 Quantitative real-time PCR (qRT-PCR) of BMP gene family

#### 2.7.1 Sample collection

According to previous studies (Lei et al., 2022) (Zhou et al., 2019), the gonads of *P. sinensis* were undifferentiated at 9-day and the 16-day was the critical period for gonad differentiation, under incubation conditions at a temperature of 31°C and 75% humidity. To verify the reliability of RNA-Seq results and explore the functions of Bmp gene family members initially, the relative expression of the Bmp gene family members was detected in the gonads of 9-day, 16-day male and female embryos and in 6-month-old male and female gonads by qRT-PCR.

All experimental procedures were performed in accordance with the regulations for animal care of the Pearl River Fisheries Research Institute (Guangzhou, China). All experimental *P. sinensis* and embryos were obtained from Caixing Industrial Co. (Huizhou, China). According to the sample collection method of Lei et al. (2022), embryos were collected on 9 and 16-day and gonads (including kidneys and gonads) were rapidly isolated under a light microscope, and stored in liquid nitrogen for RNA extraction. According to the Tissue DNA Extraction CZ Kit (Mabio, China) instructions, the rest of the tissue was used as DNA extraction to identify the sex of the embryos by PCR (Li et al., 2020). Three 6-month-old male and female *P. sinensis* were anesthetized using 0.05% MS-222 (20 mg/kg, Sigma, MO, United States) by intraperitoneal injection and sacrificed. Subsequently, testes and ovaries were quickly collected, and stored in liquid nitrogen for RNA extraction.

#### 2.7.2 RNA extraction, cDNA synthesis and quantitative real-time PCR (qRT-PCR)

Total RNA extraction of all samples was performed according to the RNAiso Plus (Takara, Beijing, China) instructions. After RNA quality testing by RNA electrophoresis (Bio-Rad, PowerPacTM, CA, United States) and NanoDrop 2000 Spectrophotometer (Thermo Fisher, NanoDropOne, MA, United States), cDNA synthesis was performed following the protocol of the reverse transcription kit (Takara, Beijing, China).

Follow the instructions of iTaq Universal SYBR Supermix (BIO-RAD, CA, United States) for qRT-PCR. All the primers were designed based on the nucleotides of *P*. *sinensis* BMPs family genes on NCBI, the *Ef1a* gene was chosen as a reference gene to calculate the relative expression of the target genes since it showed a stable expression pattern in *P. sinensis*. The qRT-PCR volume was 20 μL, including 10 μL of SYBR Supermix, 1 μL of cDNA, 1 μL of each primer (2 μM) and 7 μL of nuclease-free water. The PCR cycling conditions for all target genes and *Ef1a* were as follows: 95°C for 10°min; 40 cycles of 95°C for 15 s, 55°C–60°C for 15 s, and 72°C for 15 s; and melting curve analysis at 95°C 15 s, 60°C for 60 s, and 95°C 15 s. Each sample was analyzed in triplicate. The sequences of the primers for the target genes and reference genes are given in Table 1. Transcript expression levels were analyzed using the 2^−ΔΔCT^ method (Livak and Schmittgen, 2001). Differential expression analysis was performed by ANOVA (Momen et al., 2001). The results are presented as the means ± SEM of three replicates, and the statistical significance is represented by a *p*-value <0.05.

**TABLE 1:** The primers sequences of qRT-PCR.

Primer name	Sequence (5′–3′)	Product size (bp)
*Bmp2*-F	AAC​GCC​ACA​AAT​CCA​GTT​GC	220
*Bmp2*-R	GGA​ACG​CAG​CAA​GCT​TTA​GG	
*Bmp2l*-F	ATC​ACT​TCT​GCC​AGG​TGA​GC	128
*Bmp2l*-R	TCT​TCC​ATC​CAG​TCA​CGC​AC	
*Bmp5l*-F	AGA​GCT​TCC​CAT​TTC​CTC​CC	108
*Bmp5l*-R	TCT​GAT​GTT​CGC​CAA​GAC​CT	
*Bmp2bl*-F	GTG​TTG​GCA​TTG​GAT​GAG​CT	183
*Bmp2bl*-R	ACT​CTG​GGT​GCA​TCT​GTT​CA	
*Bmp3*-F	ACT​CTC​CAG​TTT​GAC​GAG​CA	238
*Bmp3*-R	CAA​CTG​CTC​TCA​CGA​TGC​TC	
*Bmp5*-F	GCC​TCA​ATC​AAA​GCA​GCC​TT	233
*Bmp5*-R	AAT​CCT​GCC​ATC​CCA​AGT​CA	
*Bmp6*-F	GTC​CTT​ACG​ACA​AGC​AGC​CT	119
*Bmp6*-R	CTG​AGT​GGA​GCG​GTT​TCG​AT	
*Bmp7*-F	GAC​AGC​AAC​TTC​CTC​ACG​GA	266
*Bmp7*-R	ATT​CCC​GAT​GGT​GAT​ACC​GC	
*Bmp8a*-F	CGA​AGG​CTG​GTT​GGT​TTT​CA	237
*Bmp8a*-R	TGC​CTC​TTC​CTT​AGC​TGC​TT	
*Bmp10*-F	TCC​TGA​AGA​CGC​TGA​ACC​TT	117
*Bmp10*-R	ATG​GCA​TTG​AGG​TCC​TGT​CA	
*Bmp15l*-F	CGT​GGT​GCA​GAA​CTT​TGT​CA	125
*Bmp15l*-R	CTT​TGT​AGA​GGA​TGC​TGC​CG	
*Ef1a*-F	ACT​CGT​CCA​ACT​GAC​AAG​CCT​C	337
*Ef1a*-R	CAC​GGC​GAA​CAT​CTT​TCA​CAG	

## 3 Results

### 3.1 Identification of the members of BMP gene family in the *P*. *sinensis* genome

A total of 11 BMP gene family members named *Bmp2* (XM_006136012.3), *Bmp2l* (XM_006123882.3), *Bmp2lb* (XM_014571013.2), *Bmp2bl* (XM_006117827.3), *Bmp3* (XM_006110911.3), *Bmp5* (XM_006110971.2), *Bmp6* (XM_025179361.1), *Bmp7* (XM_006125345.3), *Bmp8a* (XM_025179496.1), *Bmp10* (XM_006110793.3), and *Bmp15l* (XM_025185441.1) were initially identified within the genome of *P*. *sinensis*. Bmp6 was discarded due to the absence of a complete protein sequence, and 10 members of the BMP gene family were finally selected for subsequent analysis.

### 3.2 Phylogenetic analysis of identified BMP genes

**FIGURE 1:**
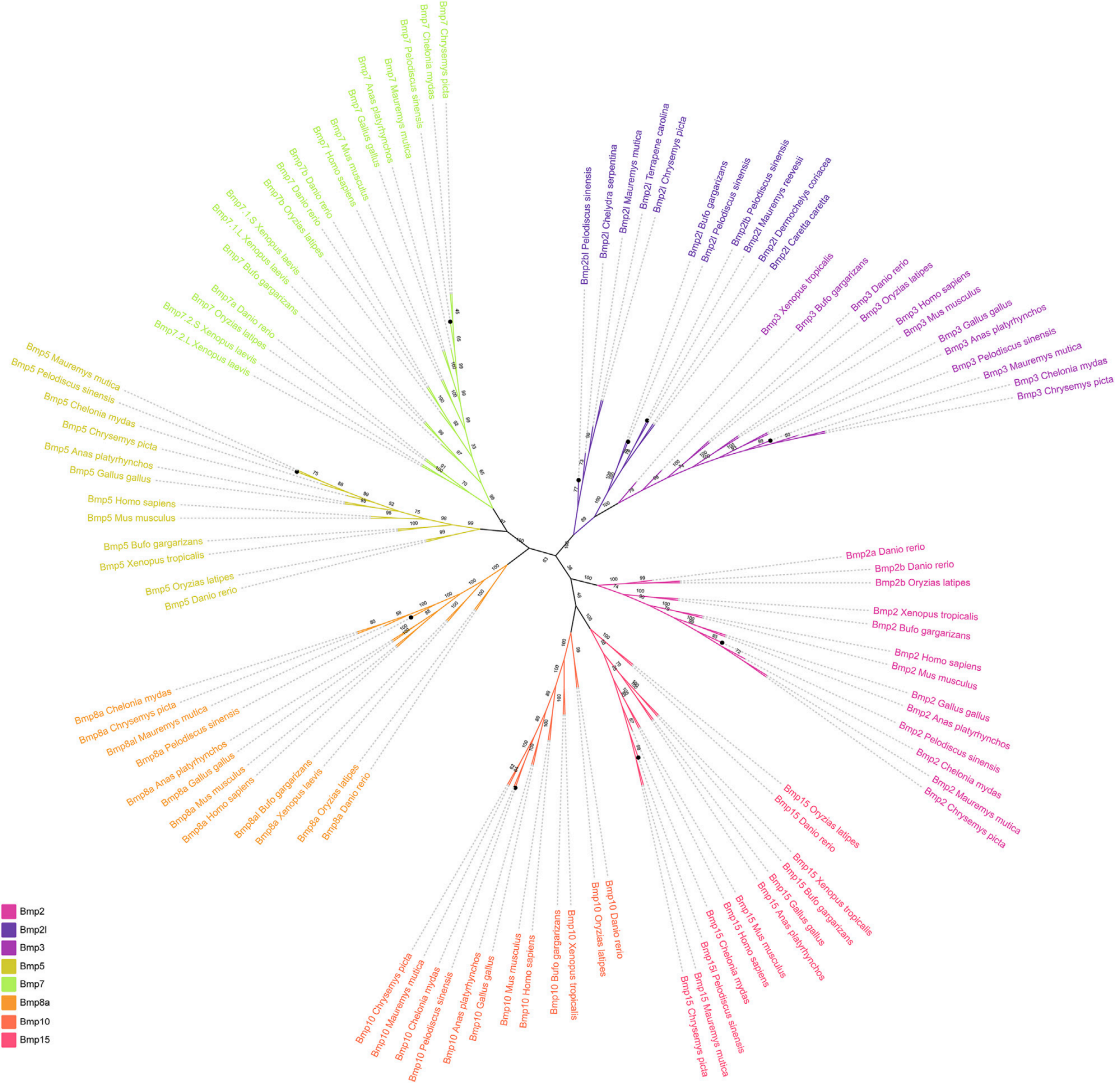
Phylogenetic tree of the BMP gene family among different vertebrates. Different colors represent different BMP gene subfamilies. The black circles indicate the BMP genes in *P. sinensis.*

To investigate the phylogenetic relationships of BMP genes in *P*. *sinensis*, an unrooted phylogenetic tree was constructed (Figure 1). The BMP genes of *P*. *sinensis* were clustered into eight subfamilies including Bmp2, Bmp2l, Bmp3, Bmp5, Bmp7, Bmp8a, Bmp10, and Bmp15. The majorities of BMP genes in *P*. *sinensis* were first clustered with turtles, followed by frogs and birds. Significantly, *Bmp2l*, *Bmp2l,b* and *Bmp2bl* of *P*. *sinensis* were clustered into one subfamily, suggesting that the function of these genes may have changed during the evolution and development of *P*. *sinensis* compared to the members of Bmp2 subfamilies.

### 3.3 Collinear analysis

**FIGURE 2:**
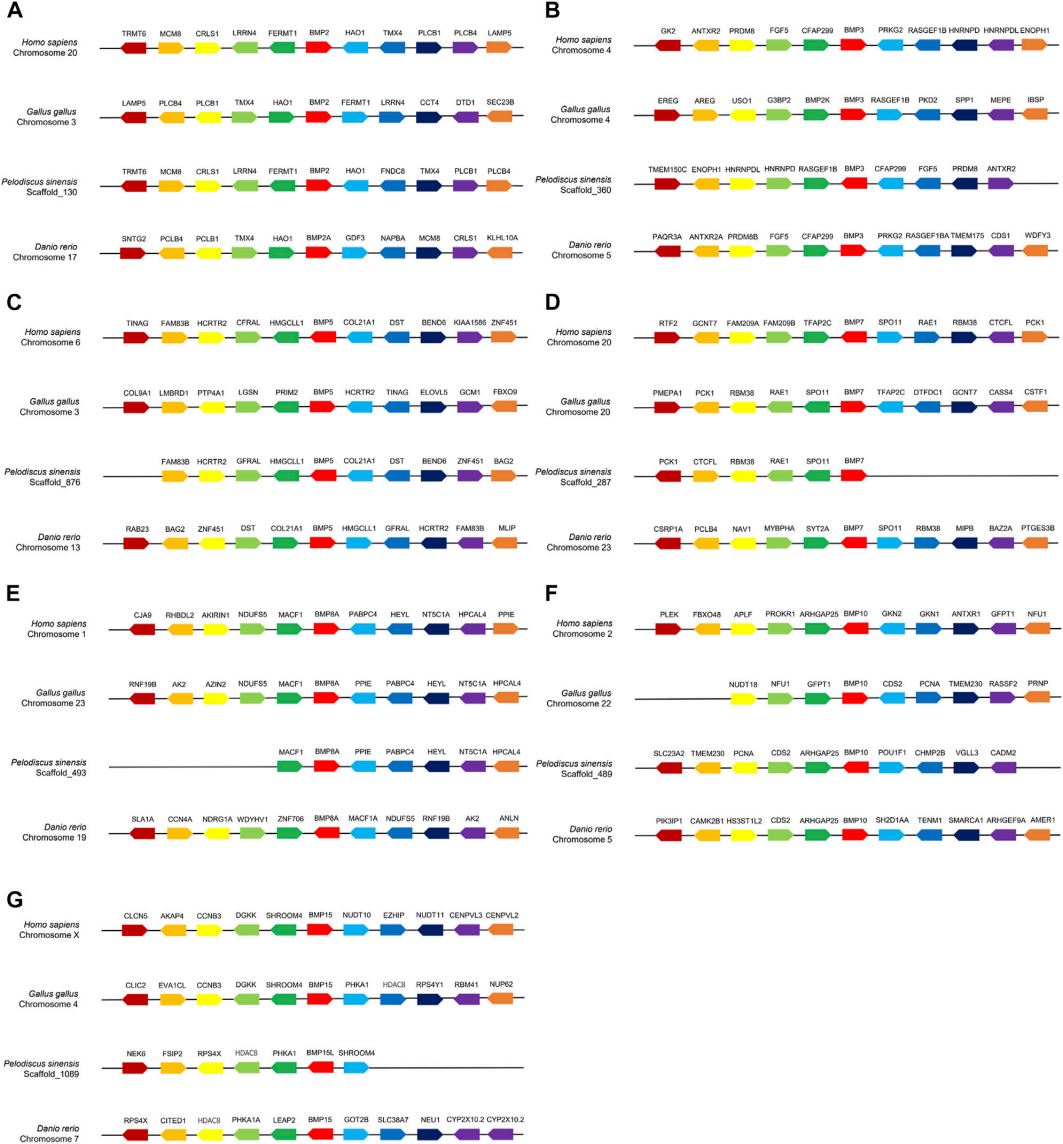
Collinear analysis of *Bmp2* **(A)**, *Bmp3* **(B)**, *Bmp5* **(C)**, *Bmp7* **(D)**, *Bmp8a* **(E)**, *Bmp10* **(F)**, *Bmp15* **(G)** and their adjacent genes in *Homo sapiens, Gallus gallus*, *P*. *sinensis* and *Danio rerio*. Direction of the arrows indicates gene orientation.

Comparative gene collinear analysis was performed between *P*. *sinensis, Homo sapiens, Gallus* and *Danio rerio* (Figures 2A–G)*.* The results showed that the *Hao1*-*Bmp2*-*Fermt1*-*Lrrn4*-*Crls1*-*Mcm8*-*Trmt6* block was highly conserved between *Homo* and *P*. *sinensis,* and four adjacent genes of *P. sinensis Bmp2* (*Hao1*, *Tmx4*, *Plcb1*and *Pclb4*) were identical in *Danio rerio* and *Gallus gallus* (Figure 2A). The *Bmp3*-*Cfap299-Fgf5-Prdm8b-Antxr2* block was highly conserved in *P*. *sinensis, Homo sapiens* and *Danio rerio.* Only one adjacent gene (*Rasgef1b*) was identical in *Gallus gallus* (Figure 2B). The *Fam83b- Hcrtr2*- *Gfral*- *Hmgcll1*-*Bmp5*-*Col21a1*-*Dst*-*Bend6* block was highly conserved in *Homo*, *P*. *sinensis a*nd *Danio rerio*. However, no conserved adjacent gene was found in *Gallus gallus* (Figure 2C)*.* The adjacent genes of *Bmp7* (*Spo11*, *Rae1*, *Rbm38*) (Figure 2D) and *Bmp8a* (*Macf1, Ppie*, *Pabpc4*, *Heyl*, *Nt5c1a, Hpcal4*) (Figure 2E) were highly conserved in *Gallus gallus*, *P*. *sinensis and Homo*. Two adjacent genes of *P*. *sinensis Bmp10* (*Arhgap25*, *Cds2*) were identical in *Danio rerio*, and three adjacent genes (*Cds2*, *Pcna*, *Tmem230*) were identical in *Gallus gallus*, and *P*. *sinensis Bmp10* shared only one conserved adjacent gene (*Arhgap25*) *in Homo sapiens* (Figure 2F)*. P*. *sinensis Bmp15l* shared only one conserved adjacent gene *in Homo sapiens* (*Shroom4*) and *Danio rerio* (*Hdac8*)*.* Compared with *Gallus gallus*, *P*. *sinensis Bmp15l* shared three conserved adjacent genes (*Phka1*, *Hdac8*, *Shroom4*) (Figure 2G).

### 3.4 Scaffold localization of BMP genes

**FIGURE 3:**
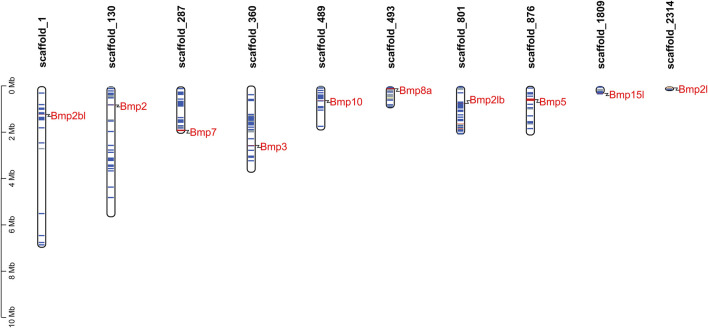
Scaffold distribution of BMP genes in *P*. *sinensis*. The blue lines represent gene density of scaffolds, and the red lines indicate higher gene density. The gene density is defined as the number of genes within a 50 kb genome.

To determine the scaffold locations of *P*. *sinensis* BMP genes, 10 members were mapped to 10 different genomic scaffolds (Figure 3). *Bmp2bl*, *Bmp2*, *Bmp7*, *Bmp3*, *Bmp10*, *Bmp8a*, *Bmp2lb*, *Bmp5*, *Bmp15l*, and *Bmp21* were located on scaffold_1, scaffold_130, scaffold_287, scaffold_360, scaffold_489, scaffold_493, scaffold_801, scaffold_876, scaffold_1809, and scaffold_2314, respectively.

### 3.5 Gene structures and conserved motifs

**FIGURE 4:**
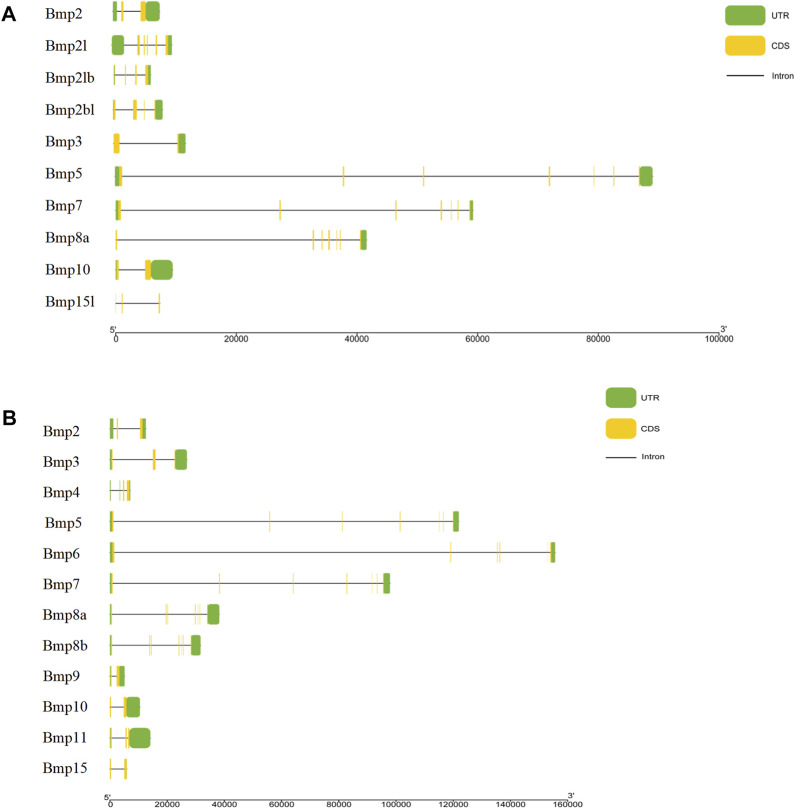
Gene structures of BMP genes. **(A)**. Gene structures of BMP genes in *P*. *sinensis*. **(B)**. Gene structures of BMP genes in *Homo.* The green boxes show untranslated regions, the yellow boxes show coding sequences, and the black lines show introns.

**FIGURE 5:**
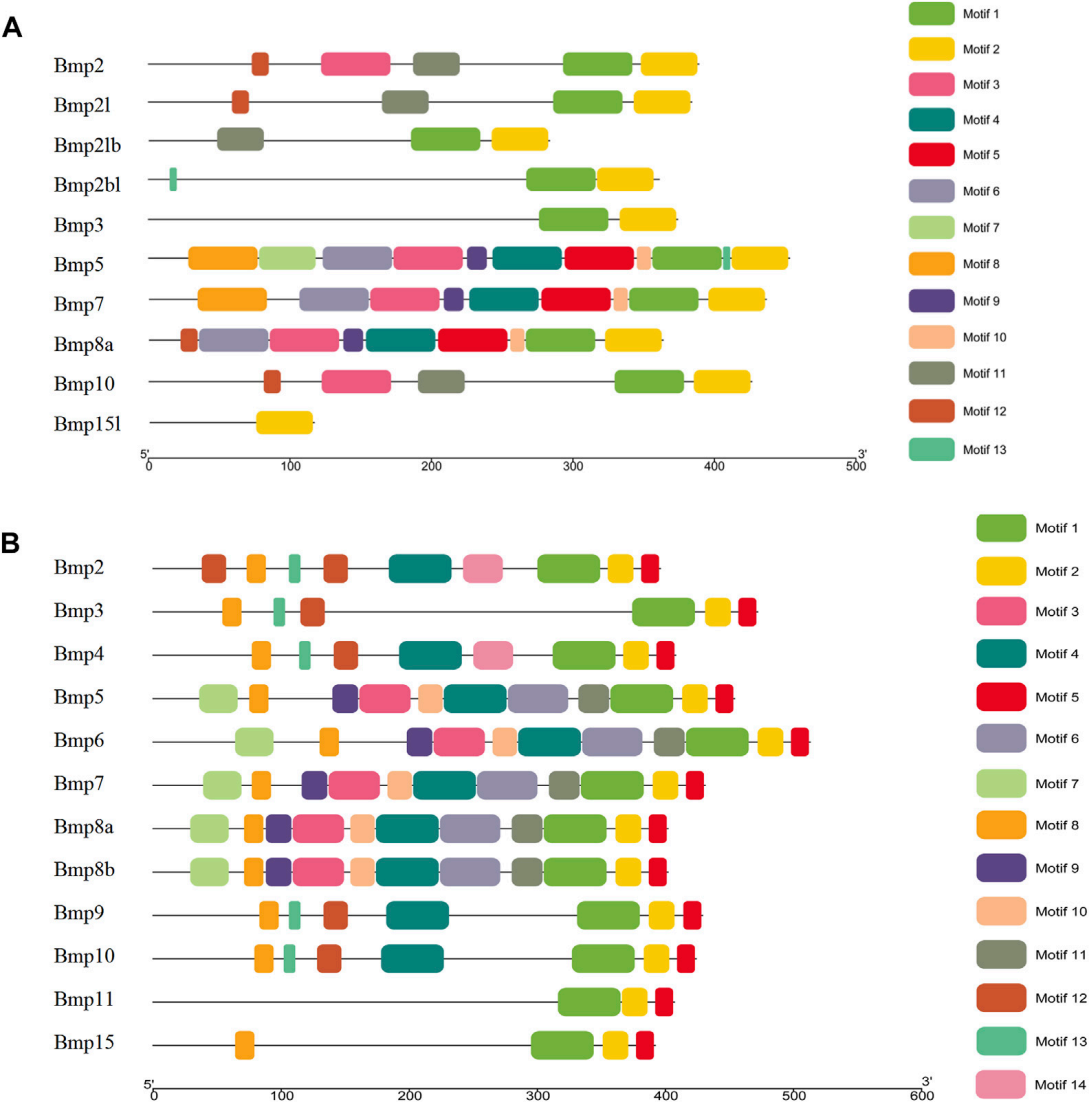
Conserved motifs of BMP genes in *P*. *sinensis* **(A)** and *Homo* **(B)**. Different colored boxes indicate different motifs; the direction of the proteins is from the N-terminal to the C-terminal.

The results of the gene structure analysis showed that the gene length, coding sequence (CDS) number, and introns of BMP genes demonstrated significant differences in *P*. *sinensis*. *Bmp2*, *Bmp3*, and *Bmp10* had the lowest numbers of CDS (2), with *Bmp5*, *Bmp7*, and *Bmp8a* having the highest CDS numbers (7). Furthermore, *Bmp8a* and *Bmp15l* lack of the 3′untranslated region (3′UTR) (Figure 4A). Similarly, in *Homo, Bmp2* and *Bmp10* had the lowest numbers of CDS (2), with *Bmp5*, *Bmp7*, *Bmp8a* and *Bmp8b* having the highest CDS numbers (7) (Figure 4B). Analysis of the conserved motifs of BMP proteins showed that 13 putative motifs Fwere unevenly distributed in *P*. *sinensis* BMP genes. The number of motifs ranged from 1 (*Bmp15l*) to 11 (*Bmp5*). Motif 1 was shared by 9 BMP genes, and motif 2 was shared by all (Figure 5A). In *Homo*, the number of motifs ranged from 3 (*Bmp15*) to 11 (*Bmp5, Bmp6, Bmp7, Bmp8a, Bmp8b*), and there are three motifs (Motif 1, Motif 2, Motif 5) shared in all BMP proteins (Figure 5B) (The sequences of all motifs are provided in Annex 2).

### 3.6 Conserved domains and multiple sequence alignment

**FIGURE 6:**
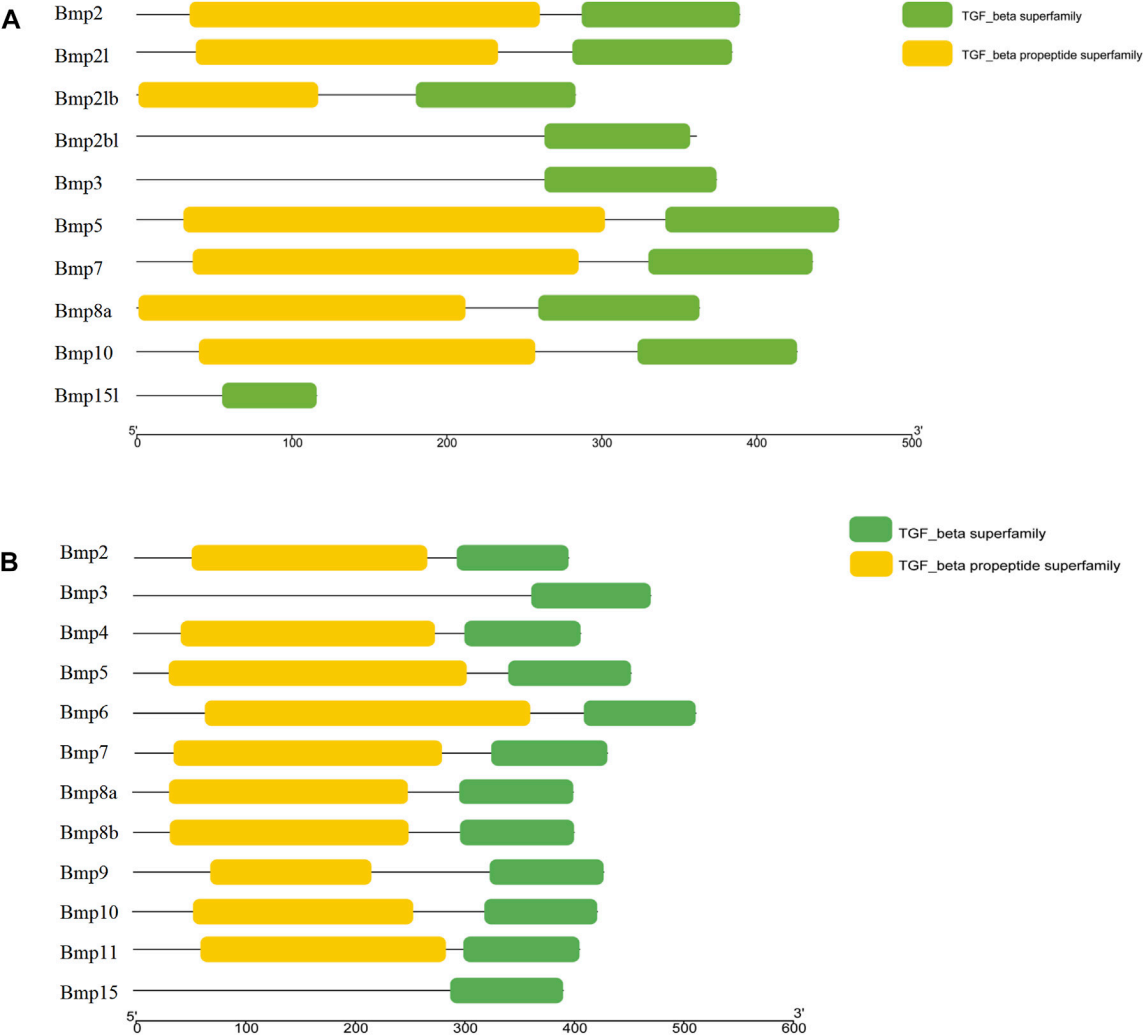
Conserved domains of BMP genes in *P*. *sinensis* **(A)** and *Homo* **(B)**. The green boxes and yellow boxes indicate the protein domains of the TGF-β superfamily and the TGF-β propeptide superfamily, respectively.

**FIGURE 7:**
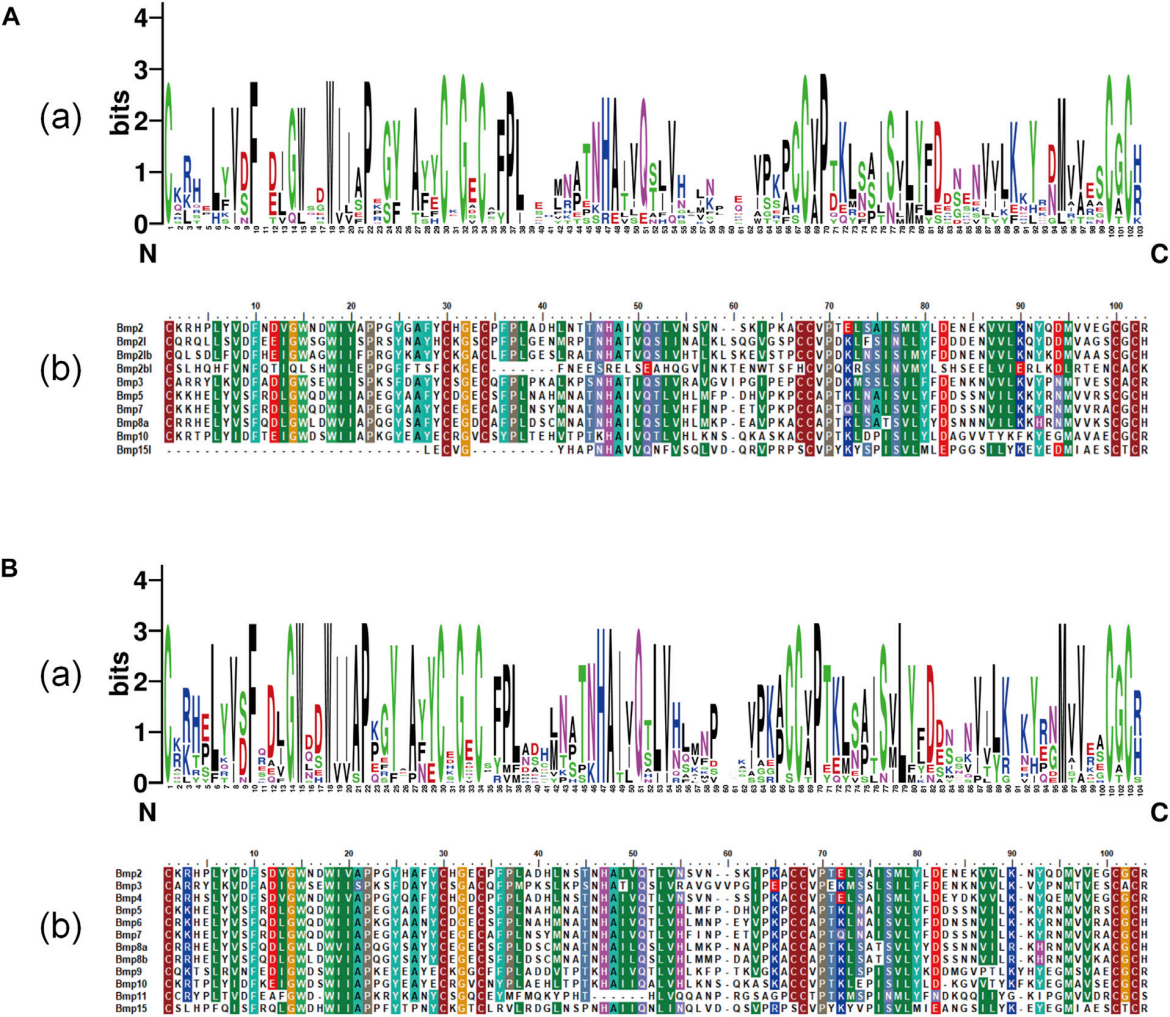
Multiple sequence alignment of TGF-β superfamily domains of BMP genesin *P*. *sinensis* **(A)** and *Homo* **(B)**. **(A)** and **(B)** show the sequence logo and amino acid sequence alignment of the TGF-β superfamily domain of BMP genes, respectively. The direction of amino acid sequences is from the N-terminal to the C-terminal.

BMP genes of *P*. *sinensis* all possessed a conserved protein domain of the TGF-*β* superfamily, and 7 proteins (70%) had two common domains, the TGF-*β* superfamily and TGF-*β* propeptide superfamily. However, Bmp2bl, Bmp3, and Bmp15l only had one domain (Figure 6A). Likewise, all BMPs in *Homo* shared the conserved protein structural domain of the TGF-*β* superfamily, and there was only one domain in Bmp3 and Bmp15 (Figure 6B). The results of multiple sequence alignment of the conserved protein domain (TGF-*β* superfamily) showed that conservative motifs of “WII”, “FPL”, “TNHA”, “CCVP”, and “CGC” were shared by almost all BMP genes in *P*. *sinensis*. However, a few conserved motifs of BMP genes have undergone mutation and evolution. For instance, the motifs of Bmp2bl and Bmp15l changed from “TNHA” to “ESRE” and “PNHA”, respectively. Further, the motif of “FPL” was missing in Bmp2bl and Bmp15l (Figure 7A). In *Home*, “WIIAP”, “FPL”, “HAIVQ”, “ISVLY”, and “CGC” were shared by almost all BMP genes (Figure 7B). In *P. sinensis* and *Homo*, the conservative motif of “CGC” was changed to “CAC” in Bmp3 and “CTC” in Bmp15, respectively.

### 3.7 Prediction of secondary and tertiary structures

**FIGURE 8:**
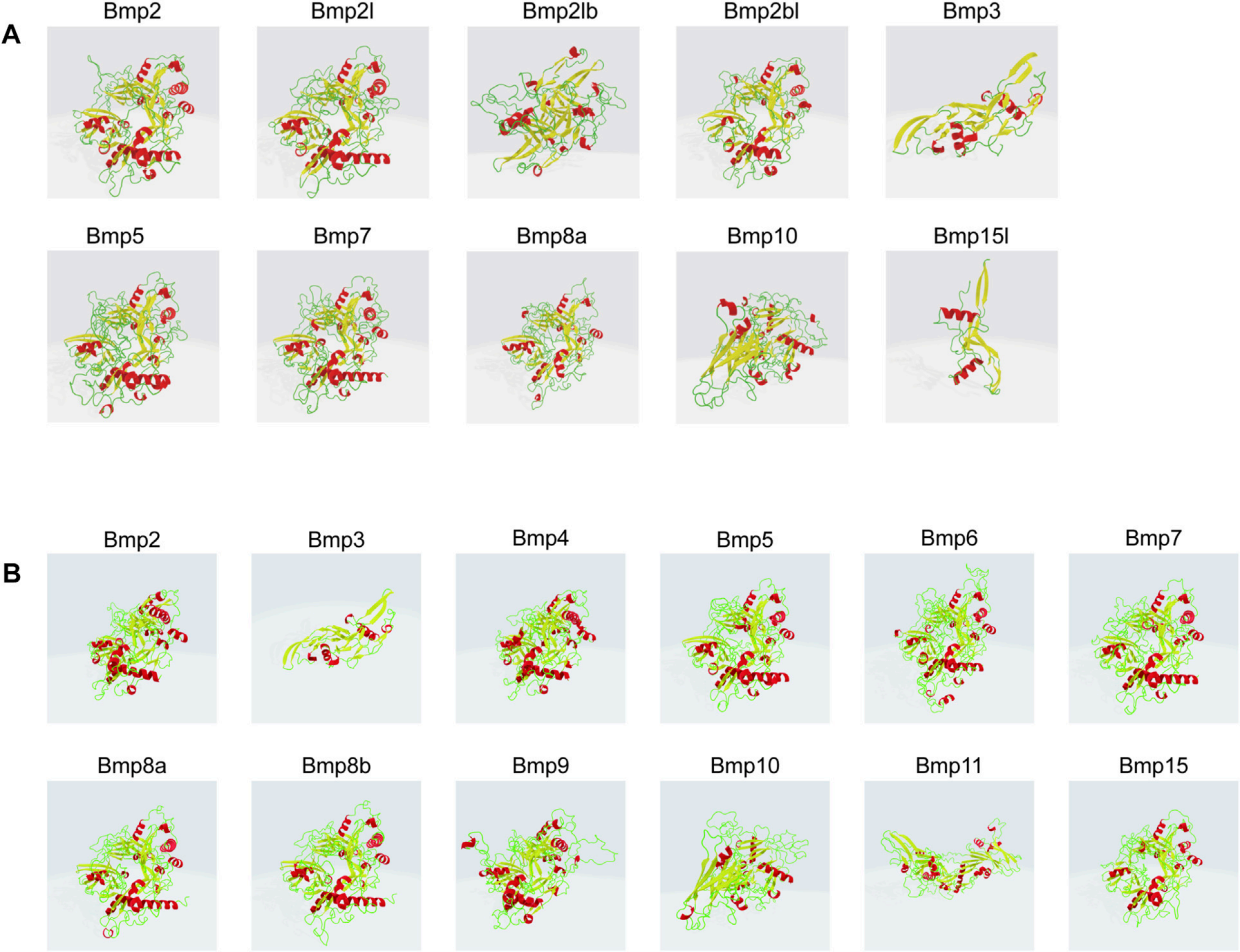
Three-dimensional modeling of BMP proteins in *P*. *sinensis* **(A)** and *Homo* **(B)**. Red indicates alpha helix, yellow represents beta turn, green shows random coil.

*P*. *sinensis* and *Homo sapiens* BMP proteins are mainly composed of helixes, beta turns, random coils and extended strands (Figures 8A, B). In *P*. *sinensis,* alpha helixes ranged from 18.18 to 39.66%, beta turns from 1.07 to 6.65%, random coils from 37.93 to 62.30%, and extended strands from 16.38 to 21.76% (Table 2). In *Homo sapiens*, alpha helixes ranged from 19.65 to 29.29%, beta turns from 1.77 to 4.09%, random coils from 51.77 to 58.87%, and extended strands from 13.77 to 20.64% (Table 3).

**TABLE 2:** The secondary structure prediction of BMP proteins in *P*. *sinensis*.

Name	Alpha helix (%)	Beta turn (%)	Random coil (%)	Extended strand (%)
Bmp2	26.22	2.57	53.73	17.48
Bmp2l	29.43	1.56	52.60	16.41
Bmp2lb	19.43	2.47	58.30	19.79
Bmp2bl	27.70	6.65	44.32	21.33
Bmp3	18.45	1.07	62.30	18.18
Bmp5	24.94	3.09	53.86	18.10
Bmp7	24.54	2.52	54.59	18.35
Bmp8a	18.18	3.58	56.47	21.76
Bmp10	30.05	3.29	48.83	17.84
Bmp15l	39.66	6.03	37.93	16.38

**TABLE 3:** The secondary structure prediction of BMP proteins in *Homo*.

Name	Alpha helix (%)	Beta turn (%)	Random coil (%)	Extended strand (%)
Bmp2	29.29	1.77	51.77	17.17
Bmp3	26.69	4.03	55.51	13.77
Bmp4	21.81	2.45	57.11	18.63
Bmp5	24.67	3.96	54.41	16.96
Bmp6	21.05	4.09	58.87	15.98
Bmp7	24.59	3.25	54.29	17.87
Bmp8a	19.65	3.23	56.97	20.15
Bmp8b	21.64	3.73	55.72	18.91
Bmp9	28.44	3.96	53.38	14.22
Bmp10	25.71	2.36	54.72	17.22
Bmp11	23.34	3.19	52.83	20.64
Bmp15	28.32	1.79	56.12	13.78

### 3.8 Prediction of transcription factors

**FIGURE 9:**
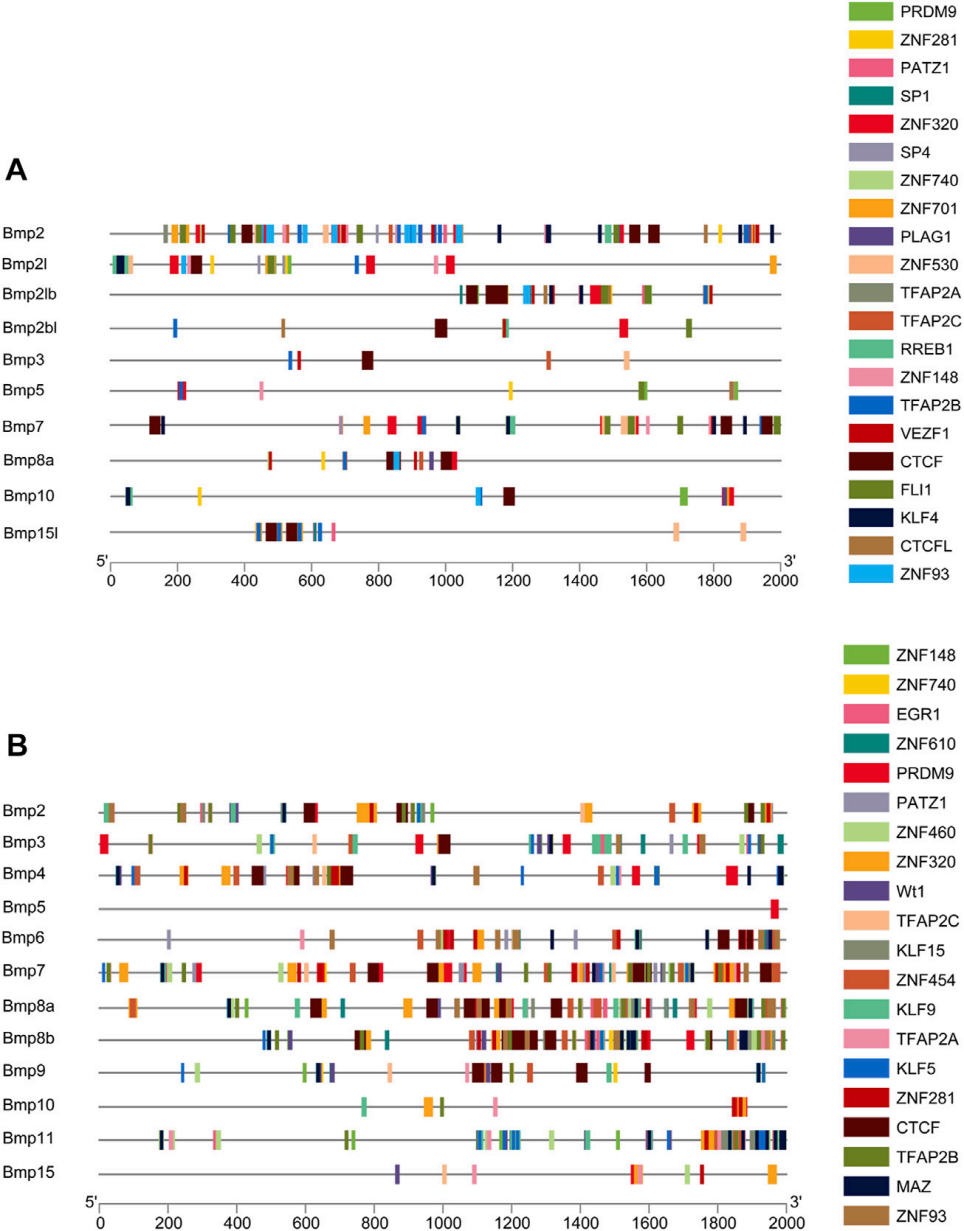
Promoter analysis of BMP genes in *P*. *sinensis* **(A)** and *Homo* **(B)**. Different colored rectangles indicate different transcription factors.

The 2-kb 5’ upstream region of *P*. *sinensis* and *Homo sapiens* BMP genes was selected to analyze transcription factors. There are 640 and 682 types unique transcription factors predicted to interact with the 2-kb promoter region of the upstream transcription start site of the BMP genes in *P*. *sinensis* (Supplementary Table S1) and *Homo sapiens* (Supplementary Table S2), respectively. Additionally, the top 20 transcription factors were chosen for visualization (Figures 9A, B). The transcription factor PRDM9 and ZNF148 were the most frequently transcription factor in *P*. *sinensis* and *Homo*, respectively. The zinc finger protein (ZFP) was associated with the broadest varieties of transcription factors of the top 20, both in *P*. *sinensis* and *Homo*. Also, the transcription factors involved in classic BMP signaling were obtained, containing SMAD2 and SMAD4 in these two species. Intriguingly, the transcription factors associated with sex differentiation including RA receptor alpha (RARA), FOXL2, and DMRT1 were also predicted in both *P*. *sinensis* and *Homo*.

### 3.9 Expression analysis

**FIGURE 10:**
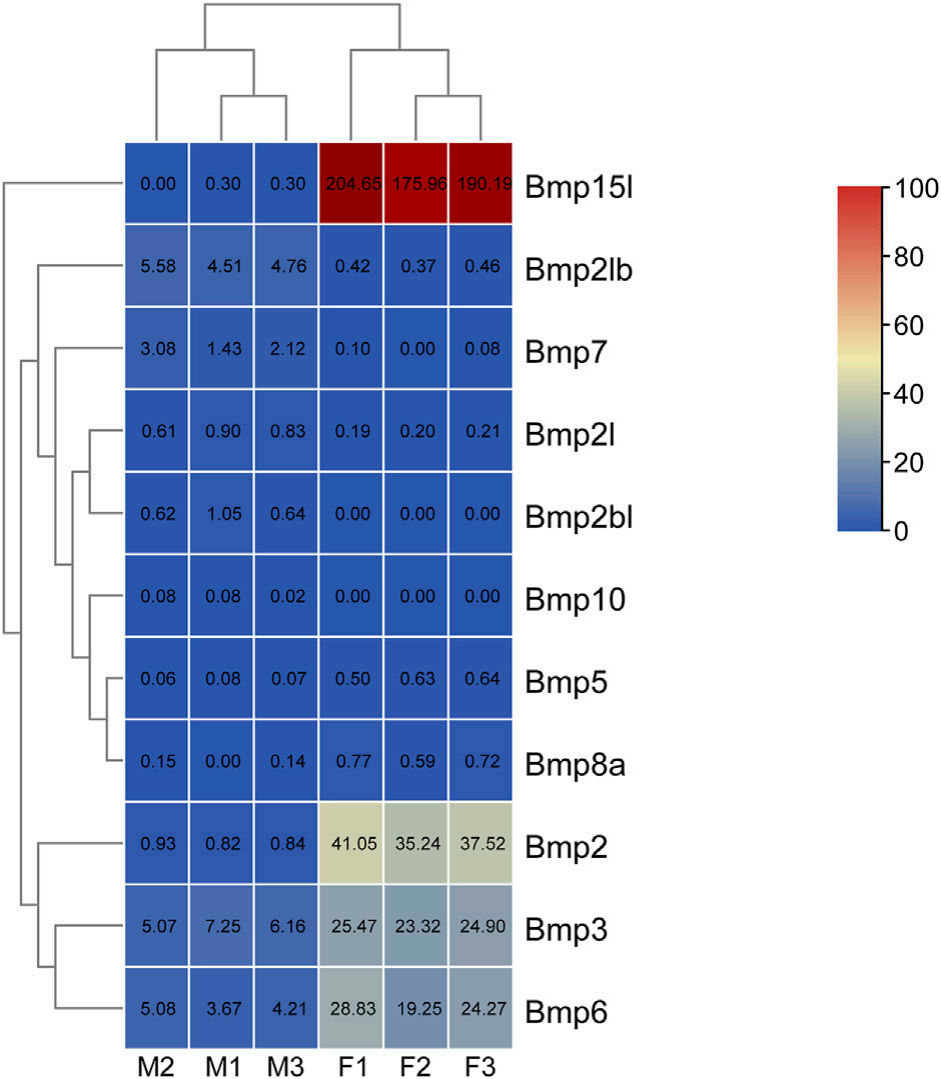
Expression analysis of BMP genes in *P*. *sinensis*. The expression levels of BMP genes are presented with FPKM values. The color scale ranges from 0 to 100, red indicates high expression, and blue indicates low expression. F1, ovary1; F2, ovary2; F3, ovary3; M1, testis1; M2, testis2; M3, testis3.

To study sexual dimorphisms in the expression profiles of BMP genes in *P*. *sinensis* male and female gonads, we analyzed transcriptome data of the early developmental gonads. As shown in Figure 8, *Bmp2*, *Bmp3,* and *Bmp15l* were strongly expressed in the ovaries. The expression of *Bmp5, Bmp6* and *Bmp8a* in ovaries was higher than in testes (8 times, 6 times and 7 times, respectively), and *Bmp2l*, *Bmp2lb*, and *Bmp7* in testes were expressed more than in ovaries (8 times, 11 times, and 37 times, respectively). Nevertheless, *Bmp2bl* and *Bmp10* were barely expressed in ovaries (Figure 10).

**FIGURE 11:**
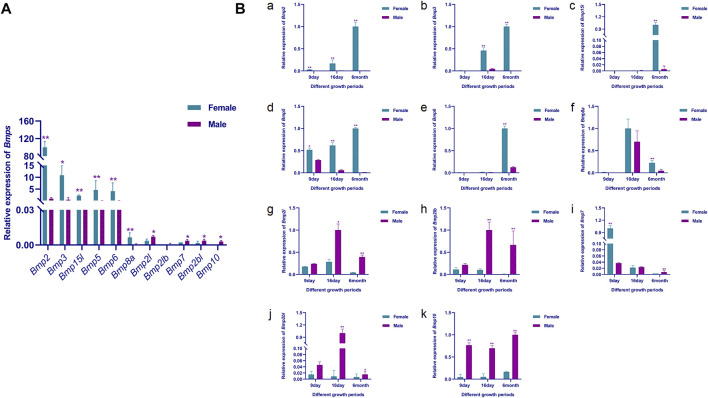
qRT-PCR validation results. **(A)**. Relative expression of BMPs in the male and female gonads of the 6-month-old *P. sinensis*. **(B)**. Relative expression of BMPs in male and female gonads at different developmental periods **(A–K)** Represent the relative expression of *Bmp2*, *Bmp3*, *Bmp15l*, *Bmp5*, *Bmp6*, *Bmp8a*, *Bmp2l, Bmp2lb*, *Bmp7, Bmp2bl and Bmp10* in gonads at different developmental stages, respectively.11 Tables.

To further validate the RNA-Seq results and initially explore the expression of the Bmp gene family in male and female gonads at different developmental stages, the expression patterns of the BMPs in male and female gonads at 9-day, 16-day and 6-month-old was examined by qRT-PCR. Consistent with the RNA-Seq results, the relative expression of *Bmp2*, *Bmp3*, *Bmp15l*, *Bmp5, Bmp6* and *Bmp8a* were extremely significantly higher in the ovary than in the testis of six-month-old *P*. *sinensis* (*p* <0.05). In contrast, *Bmp2l, Bmp2lb, Bmp7, Bmp2bl* and *Bmp10* were male-biased genes in 6-month-old *P*. *sinensis* by qRT-PCR, which was matched with the RNA-Seq results. Moreover, the expression of these male-biased genes was very weak compared with female-biased genes (Figure 11A). As the gonads develop, the expression of *Bmp2, Bmp3* and *Bmp5* in the ovary gradually increases. Interestingly, the expression of *Bmp2* and *Bmp5* showed a significant difference at 9-day (*p* <0.05). The relative expression of *Bmp2l*, *Bmp2lb* and *Bmp2bl* was highest at 16-day in testes from different developmental periods. Another interesting finding was that on 9-day, the relative expression of *Bmp7* was extremely significantly higher in female gonads than in male (*p* <0.01), which is the exact opposite of the expression pattern of *Bmp7* in six-month-old gonads (Figure 11B).

## 4 Discussion

BMPs are key factors in bone formation and differentiation (Lademann et al., 2020), but their roles in sex differentiation are less reported, especially in turtles. In the present study, we successfully identified 11 members of the BMP gene family in the *P*. *sinensis* genome. In contrast to other species, *P*. *sinensis* is missing some BMP genes, such as *Bmp8b*, and *Bmp9* (Ehata and Miyazono, 2022). It remains unclear whether this is a loss of evolution or due to a lack of genomic data in *P*. *sinensis*. The subfamily of Bmp2l seems to be unique to turtles and frogs. They are distinct from the subfamily of Bmp2 and clustered individually as a branch. Conserved regions of genes can be identified through gene distribution and permutation (Xu et al., 2022). Collinear analysis demonstrated that adjacent genes block of *Bmp5* were the most conserved in *P*. *sinensis, Homo* and *Gallus gallus*, implying that *Bmp5* may be regulated by similar mechanisms and play similar functions in these species*.*

Gene duplication occurs in three main forms including tandem, fragment, and genome duplication, which are the primary drivers of gene family expansion (Lee et al., 2015) and provide the raw materials for the evolution of gene function (Jin S. et al., 2022). The presence of two or more genes within 200 kb on a chromosome means that there are tandem duplicate gene pairs in the region (Holub, 2001). Nevertheless, we found 10 BMP genes that were unevenly distributed on 10 different genomic scaffolds, showing that the expansion of BMP gene family was not through tandem duplication in *P*. *sinensis*. Additionally, they shared distinct gene structures, amino acid sequences, protein structures, conserved motifs, and domains, suggesting that they might have functional differences. Previous study showed that BMPs had a predomain (Mulloy and Rider, 2015), of the TGF-*β* propeptide superfamily, which was also found in Bmp2, Bmp2l, Bmp2lb, Bmp5, Bmp7, Bmp8a, and Bmp10.

Concurrently, transcription factors can bind to cis-acting elements to regulate gene expression and participate in developmental processes (Spitz and Furlong, 2012). Among the 640 predicted transcription factors in *P*. *sinensis*, the classical BMP signaling pathway members were included. Classical BMP signaling is a highly conserved cascade reaction involving BMP ligands, two types of receptors (type I and type II), and the signal transduction molecules Smads, and this signaling regulates multiple biological events (Massagué, 2000; Shi and Massagué, 2003). More importantly, the transcription factors related to sex differentiation were identified in *P*. *sinensis* and *Homo*. RA functions through its receptor RARA (Huebner et al., 2018). RA is critical for the production of oocytes and sperm in mammals (Endo et al., 2019). In *Mauremys mutica*, the expression levels of some meiosis genes in ovarian cells were significantly increased by RA treatment (Liu et al., 2022). Exogenous RA treatment activates *Dmrt1a* and *Amh*, thereby inhibiting germ cell differentiation, and genetic knockout of the RA degrading enzyme *Cyp26a* promoted meiosis and oogenesis in medaka (Adolfi et al., 2020). FOXL2 and DMRT1 are the critical members of the feminization and masculinization pathways, respectively (Li and Gui, 2018). In mammals, deletion of DMRT1 led to male to female sex reversal (Huang et al., 2017), and knockout of FOXL2 induced the transformation of ovarian cells into testis-like cells (Chen et al., 2022). In *P*. *sinensis*, *Dmrt1* (Sun et al., 2017) and *Foxl2* (Jin L. et al., 2022) have been shown to be essential genes for male and female differentiation, respectively. In fish, functional deficiency of DMRT1 resulted in testicular degeneration and proliferation of steroidogenic cells, and loss of FOXL2 caused complete sex reversal in females (Li et al., 2013). Likewise, similar experimental results were also presented in birds (Smith et al., 2009; Luo et al., 2020) and other reptiles (Ge et al., 2018).

Furthermore, it is well-known that the expression pattern of genes is closely related to their function (Zhang et al., 2022b). BMP genes presented a sexually dimorphic expression pattern in the male and female gonads of *P*. *sinensis*. As female-biased genes, the expression of *Bmp2*, *Bmp3* and *Bmp5* increased with ovarian development, and showed differential expression between males and females at 16-day, implying that these genes may be closely associated with ovarian differentiation and development in *P*. *sinensis*. In mouse, *Bmp2* plays an important role in ovarian development, *Foxl2* and *Bmp2* act cooperatively to regulate *Follistatin* gene expression during ovarian development (Kashimada et al., 2011). *Bmp2* interacts with the downstream effector *Zglp1* to determine the oogenic fate of mice (Nagaoka et al., 2020). Loss of *Sox9* enhanced the expression of *Bmp2* and follistatin (Chaboissier et al., 2004). In the chicken embryo, *Bmp3* is preferentially expressed in the developing ovary (Carré et al., 2011), and in *Muscovy ducks*, *Bmp3* induced differentiated gonads to develop as females (Bai et al., 2020). Research has shown that *Bmp5* might play a fully paracrine role in rodent ovarian folliculogenesis (Magro-Lopez and Muñoz-Fernández 2021). Simultaneously, *Bmp6*, *Bmp8a* and *Bmp15l* were significantly more expressed in the ovaries than in the testes of six-month-old *P*. *sinensis,* and it is speculated that these three genes may play an important role in the physiological activities of the ovaries. *Bmp6* promotes 17β-estradiol and progesterone secretion in goat ovarian granulosa cells (Song et al., 2022). In rat, that *Bmp8* may promote female fertility by inducing cumulus cell expansion through the Smad1/5/8 pathway (Wu and Luo 2014). Mutation analysis demonstrated that deletion of *Bmp15* caused a failure of oocytes to enter early oogenesis (Kossack and Draper, 2019). In *Cynoglossus semilaevis*, *Bmp15* was significantly expressed in female gonads, and knockdown of *Gdf9* induced upregulation of *Bmp15* (Shi et al., 2022). The 16-day of embryonic development is a critical period for the sex differentiation of *P*. *sinensis* (Zhou et al., 2019). Interestingly, the expression of *Bmp2l, Bmp2lb* and *Bmp2bl* was highest in testes at 16-day, suggesting that these genes may be involved in the sex differentiation of *P*. *sinensis.* The expression pattern of *Bmp7* in gonads at different developmental stages indicates that *Bmp7* may play different roles in gonads at different developmental periods. *Bmp7* expression was significantly higher in females than in males at 9-day, suggesting that *Bmp7* is involved in female-related physiological activities before gonadal differentiation. In addition, the expression of *Bmp7* in the testis was significantly higher than that in the ovary in 6-month-old gonads, suggesting that *Bmp7* may be involved in physiological activities related to testicular development or spermatogenesis in *P*. *sinensis*. In chickens, *Bmp7* is specifically expressed in the ovary (Hoshino et al., 2005). In mouse, *Bmp7* regulates germ cell proliferation in fetal gonads (Ross et al., 2007). *Bmp7* was a downstream effector in the androgen signaling pathway of medaka and participated in the development of sex characteristics (Ogino et al., 2014). Mutation of *Bmp7* accentuated the spermatogenic defects caused by the mutation of *Bmp8a*, implying that *Bmp7* plays a role in spermatogenesis *via* a signaling pathway similar to that of *Bmp8a* (Zhao et al., 2001). Accordingly, the differential expression patterns of BMP genes in male and female gonads suggest a potential role in sex differentiation in *P*. *sinensis* and might function through the above transcription factors.

## 5 Conclusion

We performed an initial characterization of BMP genes to explore their evolution and functions, especially in sex differentiation, in *P. sinensis*. Our study not only provides complete data of BMP genes in the *P*. *sinensis* genome but also offers a novel idea to study the regulatory mechanisms of sex differentiation in *P*. *sinensis*, even in other turtles.

## Data Availability

The datasets presented in this study can be found in online repositories. The names of the repository/repositories and accession number(s) can be found in the article/Supplementary Material.
